# Using EEG-Guided Basket and Umbrella Trials in Psychiatry: A Precision Medicine Approach for Cognitive Impairment in Schizophrenia

**DOI:** 10.3389/fpsyt.2018.00554

**Published:** 2018-11-19

**Authors:** Yash B. Joshi, Gregory A. Light

**Affiliations:** ^1^Department of Psychiatry, University of California, San Diego, La Jolla, CA, United States; ^2^VISN-22 Mental Illness, Research, Education and Clinical Center (MIRECC), VA San Diego Health Care System, San Diego, CA, United States

**Keywords:** schizophrenia, mismatch negativity, biomarker, clinical trial, cognitive impairment

## Abstract

Due to advances over the last several decades, many fields of medicine are moving toward a precision medicine approach where treatments are tailored to nuanced patient factors. While in some disciplines these innovations are commonplace leading to unique biomarker-guided experimental medicine trials, there are no such analogs in psychiatry. In this brief review, we will overview two unique biomarker-guided trial designs for future use in psychiatry: basket and umbrella trials. We will illustrate how such trials could be useful in psychiatry using schizophrenia as a candidate illness, the EEG measure mismatch negativity as the candidate biomarker, and cognitive impairment as the target disease dimension.

## Introduction

In stark contrast to our growing understanding of mental illnesses, diagnoses and treatments heavily rely on the clinical interview rather than direct, reliable assays of brain function. The results are hardly surprising: we have not meaningfully improved clinically relevant endpoints for many serious mental illnesses in the last several decades (1). Recent advances in biomarker development, however, hold promise for ushering in a new era of precision medicine-style trials for treating psychiatric illnesses.

Biomarker-informed clinical trial approaches are becoming common in other fields of medicine [(2); for reviews see (3), (4)]. As one example, anti-neoplastic agents are currently selected not only based on what type of cancer a patient has and its stage, but also on the molecular phenotype and genetic aberrations unique to the cancer. Such biomarker-informed approaches are best exemplified by two conceptually related clinical trial designs: “basket” and “umbrella” trials (5, 6). Basket trials assess the effectiveness of a candidate drug based on the mechanism rather than the underlying cancer type. For example, a neoplastic drug which targets a specific genetic mutation would be given to cohorts, or “baskets,” of patients with cancers of different origin (i.e., prostate, breast, lung, etc.) who share molecular signatures, vastly expanding the number of patients that could benefit from such a precision intervention (7). Umbrella trials take patients with the same type of cancer, and assign them to treatment arms based on unique mutations—thus, every single arm is one spoke of the large “umbrella” of therapeutic interventions. As the prototypical example, the National Cancer Institute's MATCH trial recruits patients with advanced solid tumors, lymphomas and myelomas, performs extensive genotyping and molecular stratification, and places participants into one of over a dozen different treatment arms (8).

While psychiatry currently has no candidate biomarkers which have graduated from academic laboratories to guide treatments in real-world settings, the stage is being set for a future which successfully leverages a precision psychiatry approach. In this brief review, we provide an overview of what a precision psychiatry approach could look like using a well-validated translational electroencephalography (EEG) measure called mismatch negativity (MMN) as a candidate biomarker, and neurocognitive impairment in schizophrenia as a target disease dimension.

## Schizophrenia and neurocognitive impairment

Schizophrenia (SZ) is characterized by positive (e.g., hallucinations, delusions, etc.) and negative (e.g., avolition, diminished emotional expressivity etc.) symptoms which contribute to functional impairment. Beyond these defining symptoms of SZ, hundreds of studies have suggested that neurocognitive impairments are both core features of the illness and robust determinants of psychosocial disability (9–12). Neurocognitive deficits in SZ are broad, and include abnormalities in perceptual functioning, attention, verbal and non-verbal memory, language, and executive functioning (13). The severity of deficits on these neuropsychological domains are directly linked to diminished community functioning and impaired activities of daily living (14, 15).

Indeed, recent analyses of over 1,400 patients with chronic psychoses recruited for the multi-site Consortium on the Genetics of Schizophrenia (COGS) provided strong empirical support for a hierarchical model linking cognition with functional outcome in SZ (16). In this study, structural equation modeling was used to better understand how functional outcome in SZ could be better understood in relation to symptoms, cognition and early auditory information processing (EAIP). Interestingly, abnormalities in EAIP, as indexed by EEG biomarkers, had a direct and causal effect on cognition, which in turn directly affected negative symptoms, impacting overall functional outcome. Particularly noteworthy was the finding that abnormalities in EEG biomarkers linked to EAIP also independently affected functional outcome in SZ patients.

Neurophysiological indicators have indexed abnormalities in EAIP in SZ for several decades, and differences in EAIP in patients are prominently featured as endophenotypes in genomic studies. The above analyses confirmed that the neurocognitive impairments in SZ appear to be a core disease component, reliably able to be measured and directly-linked to the symptoms and functional outcomes. Despite this advance, decades of clinical trials testing the effectiveness of currently approved antipsychotic medications and other novel therapeutics as putative pro-cognitive agents have failed to improve cognitive symptoms in SZ in any durable, meaningful way (17, 18). The development of novel pro-cognitive treatment strategies is therefore of paramount importance but remains a critical unmet need (19). These elements provide the ground on which biomarkers can be used to guide research and clinical implementation of novel precision-medicine therapeutic strategies in SZ.

## Mismatch negativity: a neurophysiological biomarker for early auditory information processing

The usefulness of EEG measures in guiding new treatments depends on their ability to serve as biomarkers. Useful biomarkers must be accessible and measurable in preclinical models of disease; should be sufficiently well-characterized such that those biomarkers are linked to relevant underlying neural circuits and known mechanisms of dysfunction in psychiatric disease; and are able to be assessed in both healthy subjects and affected individuals. For usefulness in human trials biomarkers must be insensitive to practice or order effects, reliable, and responsive to interventions. To succeed in real-world settings, biomarker acquisition should also be scalable, low-cost, and suitable for use in multi-center studies.

All of the above criteria have been identified for biomarker development for neurocognitive impairment in SZ by a variety of expert consensus panels (20–23). The first panel, the Measurement and Treatment Research to Improve Cognition in Schizophrenia (MATRICS) initiative, agreed that there was a lack of consensus on a well-accepted instrument for measuring neurocognition in clinical trials (20), on the best molecular targets for drug development, on the optimal trial design for studies of those targets, and how regulatory agencies ought to approve and label novel agents. The outcome of this initiative identified the following criteria as desirable in an FDA-approved battery for use in clinical outcome measures: high test-retest reliability, utility as a repeated measure, relationship to functional outcome, tolerability and practicality, and responsivity to pro-cognitive therapeutics. The Cognitive Neuroscience Treatment Research to Improve Cognition in Schizophrenia (CNTRICS) initiative, launched after MATRICS, further expanded on the MATRICS criteria by adding that measures should have construct validity, be mechanistically related to relevant neural circuitry, and be measurable in animal models (21, 22).

At the time of CNTRICS, mismatch negativity (MMN) was already considered a mature neurophysiologic biomarker based on meeting the above criteria, and generally believed to be ready for widespread implementation in clinical trial studies (21, 23). In fact, as a real-world readiness demonstration, MMN has been extensively characterized in multi-center trials without the use of highly-trained specialists or centers (16, 24).

**Figure 1:**
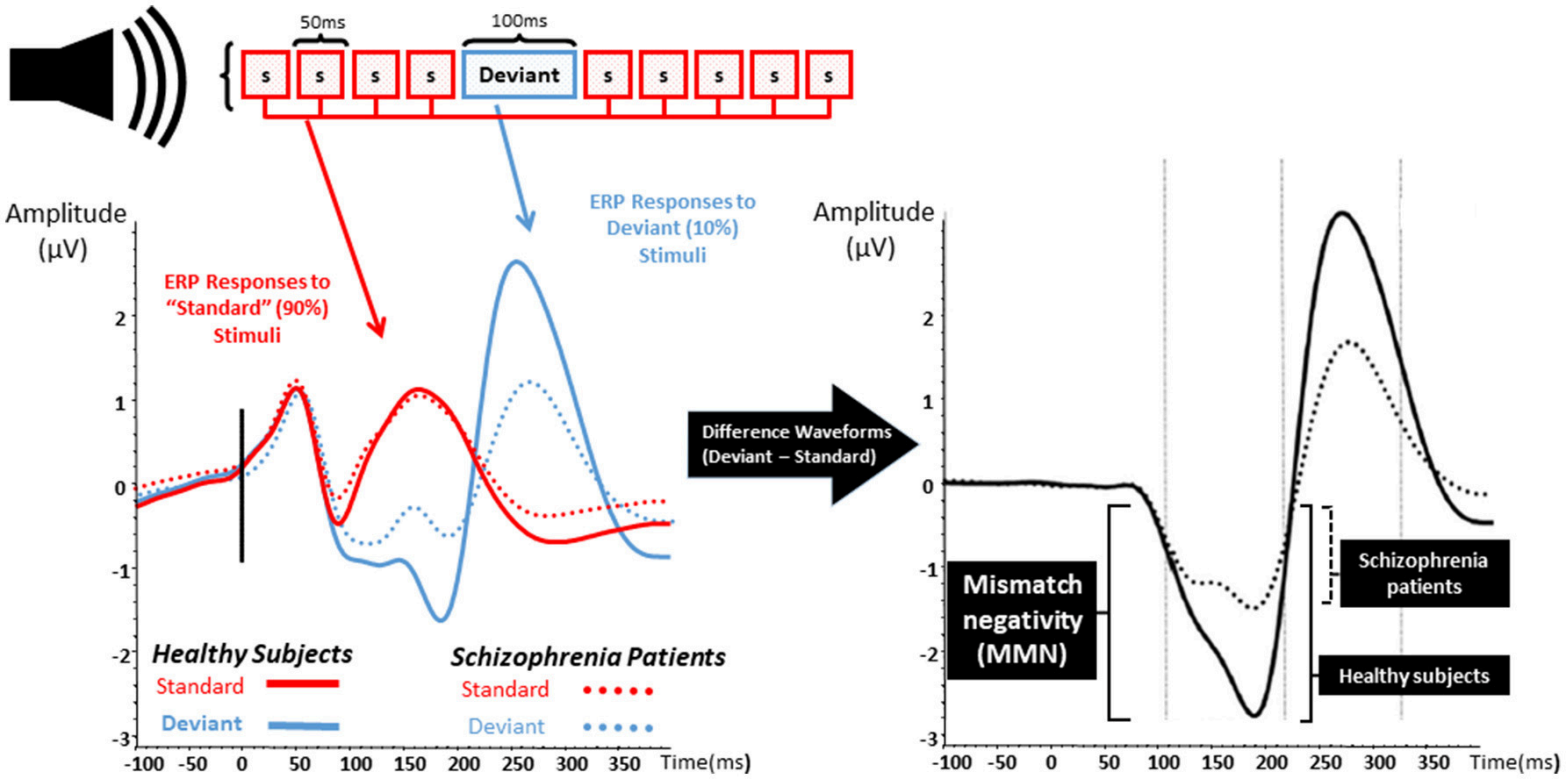
Mismatch negativity. The left graph represents the event related potentials evoked by trains of standard stimuli (S; in red) with interposed rare deviant stimuli (blue) in both healthy subject (solid lines) and patients with schizophrenia (dashed lines). The right graph represents the difference waveform, a negative inflection 100–200 ms after stimulus onset which is called mismatch negativity (MMN). Patients with schizophrenia show a reduction in the amplitude of MMN when compared to healthy subjects.

MMN is an event-related potential and a neurophysiological measure of EAIP that is evoked when a train of “standard” auditory stimuli is interrupted by an oddball or “deviant” stimulus that differs from standards as shown in Figure 1 (25–27). Differences from standard stimuli in pitch, duration, intensity, or spatial location can elicit a deviant MMN response. MMN is pre-attentive, primarily reflects an automatic response to sensory stimuli, and is able to be evoked without effort, behavioral response, or conscious awareness (26–31). After auditory deviant stimuli presentation, MMN onset begins after ~50 ms and peaks after an additional 100–150 ms (32, 33). Localization studies have consistently revealed cortical sources located in broadly distributed temporal, frontal, and parietal brain regions (34–36).

Reduction of auditory MMN amplitude was reported over two decades ago in SZ and has been replicated numerous times (37). MMN deficits are found in those with chronic psychosis (27, 37–50), in unmedicated SZ patients (29, 40, 46, 47, 51, 52), and are shown to be resistant to antipsychotics (46, 53–58). Abnormal MMN is also found in recent-onset psychosis as well as prodromal illness (30, 51, 59–66). Baseline MMN amplitude appears to be smaller in clinically high risk populations who eventually develop psychosis at follow up, and MMN in those who do not convert appears to be similar to age-matched controls (30, 51, 63). Strikingly, MMN amplitude seems to anticipate time-to-convert to psychosis—more severe MMN deficits relate to shorter time for psychosis to declare (51, 63).

Mechanistically, auditory MMN is thought to be an index of N-methyl-D-aspartate receptor (NMDA) functioning (67, 68). NMDA receptor antagonists diminish MMN in non-human primates, and ketamine, an NMDA antagonist, reduces MMN in healthy control human subjects (69–75). Lower baseline MMN is also associated with psychotic-like behavioral effects experienced by healthy subjects when exposed to ketamine (72). Furthermore, MMN has shown to be highly heritable with amplitude reductions present in asymptomatic first-degree relatives of those with SZ (76–80). MMN deficits are also found in patients with chromosome 22q deletion, which result in congenital syndromes associated with SZ-like psychoses (81).

Arguably, the most important metric of biomarker applicability in psychiatric illnesses is the ability to track functional outcome. In patients with SZ, several studies have detailed that MMN deficits are able to account for a large degree of variance in cognitive and psychosocial functioning, as well as the ability to achieve or maintain independent living (17, 34, 46, 64, 82–86).

## Biomarker-informed insights for a precision medicine approach: MMN and cognitive enhancement strategies in schizophrenia

Given the cognitive deficits observed in SZ, many studies have attempted to use pro-cognitive drugs to help attenuate this dimension of illness (87). In particular, there has been great interest in the NMDA receptor antagonist, memantine, which has been approved for use in Alzheimer's disease (88, 89).

Memantine is a non-competitive moderate affinity NMDAR antagonist (90, 91). It is thought to bind the same site as magnesium, an endogenous blocker of the NMDA receptor channel, and impedes current flow only if the NMDA receptor channel is open. Upon depolarization, memantine rapidly leaves the NMDA receptor channel. Thus, functionally, memantine is thought to block sustained and pathological activation of NMDA receptors, but not affect physiological activity. In this sense, memantine is unique from other NMDA receptor antagonists which have slower un-blocking kinetics, (i.e., ketamine, phencyclidine). Interestingly, ketamine and phencyclidine are well-known to produce psychotogenic effects but memantine does not exacerbate psychosis or cognitive deficits in antipsychotic medicated patients (92–96). This discrepancy remains an area of active investigation.

In clinical trials with Alzheimer's disease patients, memantine has been found to have a modest pro-cognitive impact (97, 98). However, clinical trials using memantine in SZ targeting cognitive impairment have been inconsistent. Meta-analyses have suggested that memantine is associated with improvement in cognitive tests such as the Mini-Mental State Exam (94). While some double blind randomized clinical trials where memantine has been added on to antipsychotic medications also report reduction in cognitive deficits, others have not, including a study which showed cognitive improvement reported at 12 weeks was lost at 52 weeks (99–101). Given these discrepancies, it has been speculated that patient factors may be obscuring signals of memantine effects on cognition in SZ. Indeed, there is evidence that suggests that within the spectrum of illnesses in chronic psychotic disorders like SZ there exist separable cognitive “biotypes” which have different profiles of cognitive impairment (102, 103). Thus, without a clearer understanding of knowing which patients with SZ are able to experience benefits of memantine, results from clinical trials using such a pro-cognitive intervention—and more broadly, all pro-cognitive interventions in SZ—are difficult to interpret.

However, recent work assessing the effect of memantine on MMN could provide insights into a precision-medicine approach (94, 96, 103, 104). For example, our group has used a double blind single-dose placebo-controlled trial assessing the effects of memantine on MMN in patients with SZ (95, 96). This study employed a within-subject cross-over design such that all participants were randomized to receive either placebo or memantine, and 7 days later, receive the other intervention, thus allowing for each subject to serve as his or her own baseline. MMN was assessed ~ 6 h after placebo or memantine ingestion, which is the approximate T_max_ of memantine, on both testing days. We found that memantine enhanced MMN in patients with SZ; since improved MMN is associated with less cognitive impairment and greater psychosocial success, this type of signal suggests that MMN could be a biomarker of treatment engagement in pro-cognitive interventions. While only a single dose of memantine would not be expected to durably improve cognition, the ability of memantine to alter MMN in a patient could signify that such an individual has the neural plasticity to benefit from pro-cognitive interventions (96, 103, 105). Indeed, not all patients in the cohort showed MMN enhancement—but, these results suggest that for future trials which aim to test the effectiveness of pro-cognitive medications, MMN malleability in response to early exposure to a putative pharmacologic agent could be important for enriching trials to maximize a therapeutic signal.

Beyond medication interventions, MMN also has the potential to predict gains in non-pharmacologic pro-cognitive interventions in SZ. For example, there has been significant interest in using targeted cognitive training (TCT) for enhancing cognition in patients with chronic psychoses (106, 107). TCT is an emerging computerized, auditory-based intervention which aims to improve EAIP through adaptive exercises with participants (105, 108). TCT is typically delivered in 1 h sessions 3–5 h a week for ~20–40 h. At the group level patients with SZ show reduction in cognitive deficits which are linked to improved functional outcomes. However, 20–40% of subjects with SZ fail to show benefit, even in some cases, after 100 h of training (108–111). A biomarker measure that would identify which patients could benefit (or, conversely, which patients have a high likelihood of not benefitting) would be critical in scaling such a pro-cognitive intervention as part of a comprehensive neurorehabilitation strategy (112). As with malleability of MMN following initial exposure to memantine, MMN also appears to be a sensitive index of the neural systems engaged by the first “dose” of TCT exercises. In this context, Perez et al. found that MMN was a sensitive index of the perceptual learning that takes place in the first hour of training, with amplitude of MMN correlating with gains in auditory perceptual learning (113). More work has better elaborated this relationship, finding that on an individual level MMN changes in the direction of normalization after 1 h of TCT predict benefit from TCT after a full course (114).

## Umbrellas and baskets in psychiatry: a possible future for clinical trials in psychiatry

With what is currently known about MMN and neurocognitive impairment in SZ, we can consider how EEG biomarkers can be used in the service of a precision medicine approach to clinical trials in psychiatry.

**Figure 2:**
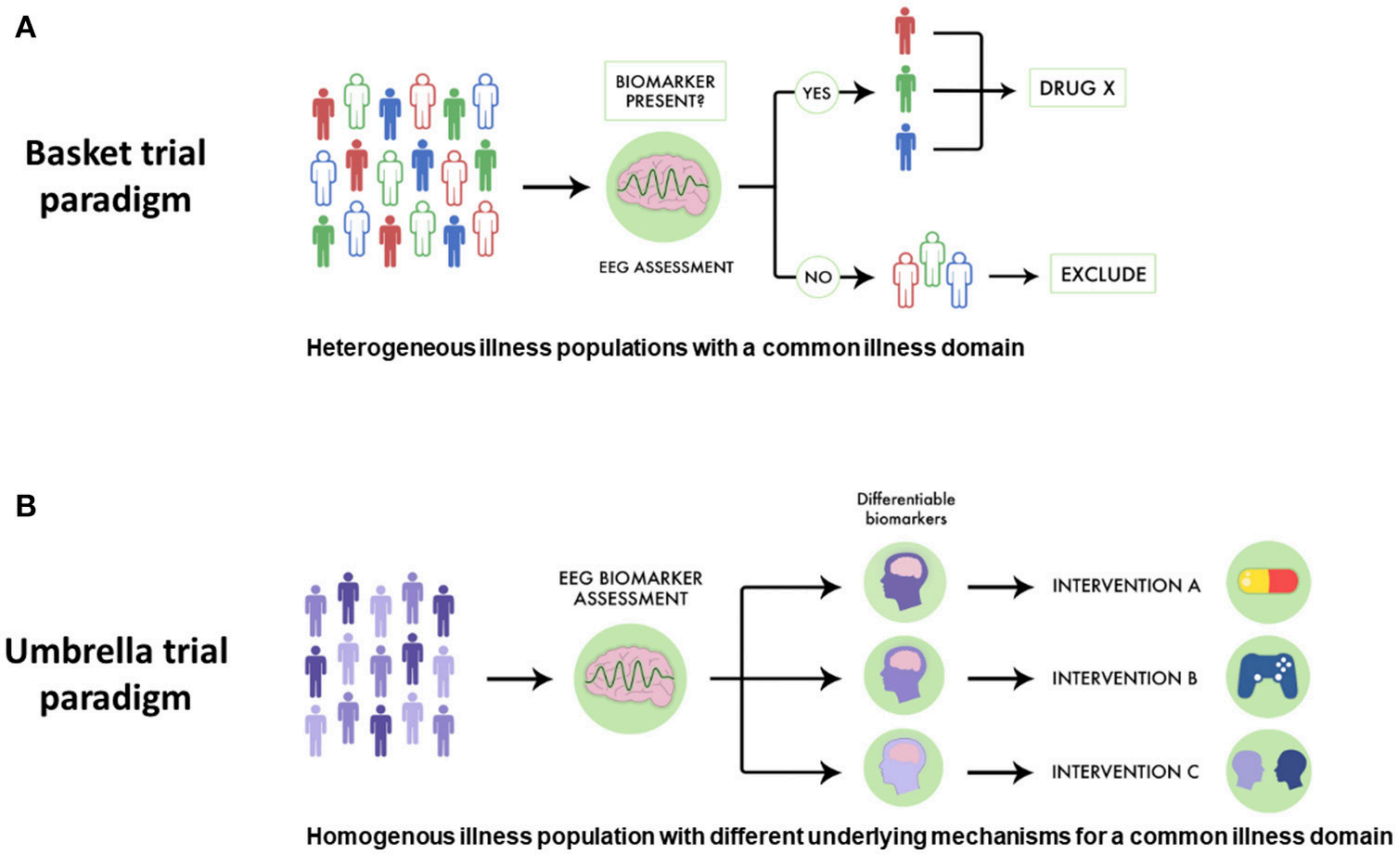
Using EEG biomarkers to design basket and umbrella trials. **(A)** Using EEG-guided basket trials could allow for different psychiatric populations who share the same disease dimension to be given a therapeutic intervention, allowing more patients to potentially benefit. In this scheme subjects are separated by different colors to denote different diseases. Those who have favorable EEG biomarker profiles are indicated by solid coloring while those are not responsive are in outline. EEG-guided basket trials have the potential to select subjects for clinical trials assessing novel drugs for optimal response. **(B)** Similarly, using EEG-guided umbrella trials could allow for patients with the same illness to be assigned to different treatments, allowing for a more specific intervention strategy. In this scheme, subjects with different biological mechanisms underlying a particular illness domain are indicated by different gradations in coloring. EEG biomarkers can be used to assess which patients may be suitable for which intervention. EEG-guided umbrella trials have the potential to improve pragmatic clinical trials assessing treatment effectiveness.

While pro-cognitive interventions for psychotic illnesses tend to focus on single diseases like SZ, cognitive impairment has been noted in related illnesses, including schizoaffective disorder and bipolar disorder with psychotic features. This parallels genetic evidence which supports a link between SZ, schizoaffective disorder and bipolar disorder. Despite this link, in traditional drug development pro-cognitive interventions are generally assessed in one population first (i.e., SZ), and then subsequent trials assess if such an intervention is useful in other related conditions. However, a basket-style precision medicine approach using EEG biomarkers could offer a more streamlined way to discover drugs targeted at transdiagnostically-related illness domains like cognitive impairment. For example, as shown in Figure 2A, a novel pro-cognitive trial testing a new Drug X could recruit patients with SZ, schizoaffective disorder and bipolar disorder and include only those who have MMN malleability. Since MMN malleability is a strong indicator of target engagement and neural plasticity, such an approach would enrich the study population to benefit from a pro-cognitive intervention. Furthermore, such a trial would test the effectiveness of a new intervention and would not necessarily be limited by traditional criteria, and have relevance across multiple illnesses (115).

Similarly, using EEG-guided umbrella designs in psychiatry would better improve pragmatic trials matching interventions to patient strengths. For example, in a SZ trial comparing different pro-cognitive interventions, positive response to particular EEG biomarkers would help stratify different treatment strategies (see Figure 2B). In such a trial, SZ patients with favorable auditory MMN malleability could receive TCT aimed to improve auditory sensory processing, while those with equivocal or poor MMN malleability could respectively receive pro-cognitive medications or specialized behavioral therapy.

These new trial designs are not without limitations. First, due to their relative novelty such designs have not yet been attempted in psychiatry, and thus there is little precedent for how these trials would be staged. Such trials require greater logistical burdens, require larger cohorts of patients, and are costlier to run. Furthermore, such trials may face barriers in recruiting enough patients with specific biomarker profiles, and experience challenges in balancing treatment arms. Both basket and umbrella trials would require new collaborative frameworks, and require nuanced statistical and administrative support.

Despite these potential limitations, using biomarkers to inform clinical trials in psychiatry holds the potential to improve our current understanding of psychiatric illness, and creates an additional way to determine the effectiveness of novel therapeutic strategies. Just as how various cancers are currently molecularly phenotyped, neurophysiologically-guided basket and umbrella trials could help “EEG-phenotype” cognitive impairment in illnesses like SZ. This precision-medicine approach would enhance the development of not only novel drugs, but also other comprehensive rehabilitation strategies in SZ like TCT.

While this mini-review has focused on neurophysiological biomarkers in SZ, the rationale described could broadly apply to other psychiatric illnesses and other types of biomarkers, including genetic and imaging biomarkers. We anticipate that as the tools of neuroscience allow us to understand complex diseases in a more nuanced way, further development of biomarker-informed precision medicine approaches to clinical trials will help further optimize matching the right treatment to the right patient.

## Author contributions

Both authors have made a substantial, direct and intellectual contribution to the work, and approved it for publication.

### Conflict of interest statement

The authors declare that the research was conducted in the absence of any commercial or financial relationships that could be construed as a potential conflict of interest. The reviewer CA and handling editor declared their shared affiliation at the time of the review.
